# Knowledge, Attitude and Practice towards Standard Isolation Precautions among Iranian Medical Students

**DOI:** 10.5539/gjhs.v4n2p142

**Published:** 2012-03-01

**Authors:** Ameneh Barikani, Ahmad Afaghi

**Affiliations:** Community medicine specialist Assistant professor of Qazvin University of Medical Sciences; Assistant professor of Qazvin University of Medical Sciences Metabolic Diseases Research Center, Qazvin, Iran E-mail: aafa2000@gmail.com

**Keywords:** Standard isolation precaution, Medical students, Knowledge, Attitude, Practice, Education

## Abstract

**Objective:**

Health care workers especially medical students are at risk of being exposed to blood-borne pathogens. Therefore, the aim of this study was to determine the knowledge, attitude and practice of medical students towards standard isolation precautions (SIP).

**Methods:**

A standardized questionnaire was completed by 148 medical students from April to July of year 2009 to seek their knowledge, attitude and practice towards standard isolation precautions in a clinical setting at Qazvin University of Medical Sciences, Iran.

**Results:**

The mean score of knowledge, attitude and practice towards standard isolation precautions were 6.8±2.1 (maximum 10), 16.6±4.2 (maximum 20), and 18.05 ± 4.5 (maximum 30) respectively. Significant differences were observed between practice of female and male (P < 0.008) and also knowledge of year 6 and year 7 students (P <0.021).

**Conclusion:**

Education on infection control based on standard isolation precaution must be stressed and barriers of appropriate practice must be removed.

## 1. Introduction

Health care workers especially medical students are at risk of acquiring infection through occupational exposure. Hospital employees can also transfer infections to patients and other office workers (Askarian, 2004). The risk of acquiring HIV by health care worker after a needle stick or other sharps injury is %5. Risk reduction must be undertaken for all blood borne pathogens through adherence to standard precautions, using personal protective equipment, appropriate use of safety devices, and providing a needle disposal system in work place (CDC, 2000). In existing reports from Iran, the incidence of acquired immunodeficiency syndrome (AIDS) is increasing rapidly, especially in intravenous drug abusers (Hatami and Mohraz, 2001). The infections rate of hepatitis B and C are 1.75 - 5% and 0.2 - 1.5% respectively (Malekzadeh, 2001). Medical students due to lake of experience in performing invasive procedures are at particular risk of exposing to blood-borne pathogens (Mohraz, 2001 & Malekzadeh, 2001). Education regarding common transmission mechanisms and preventive measures must be provided to all staff with potential exposure to blood and blood products (Garner, 1996). Several articles on the efficacy of this concept have been published (CDC, 2000; Bradley, 2003; Gershon, 1995; Kim, 1999; Koenig, 1995; Kelen, 1990). These studies demonstrated that the knowledge, attitude, and practice (KAP) of medical students towards standard isolation precautions (SIP) were not appropriate. There are limited studies in literature in relation to Iranian medical students’ KAP towards SIP. Therefore, the aim of current study was to evaluate the KAP of different groups of medical students towards undertaking SIP in clinical settings.

## 2. Material & Methods

From April to July of year 2009 a survey was conducted among 160 medical students of years 6 and 7 who had begun their clinical training in hospitals. The questionnaires were distributed by researchers with information on how to fill them out.

### 2.1 Questionnaire Design

The questionnaire included demographic characteristics of students and 10 questions in each category of knowledge, attitude and practice with regard to standard isolation precautions (Askarian, 2004). One open question also included to describe the barriers to appropriate practice. The pretest conducted on a random sample to ensure that the questionnaire was practicable and valid. Validity was assessed by infection control expert. Internal consistency coefficient (Cronbach’s alpha) was 0.82. Respondents’ knowledge, attitudes and practices with regard to standard precautions were measured. Knowledge questions had 3 possible answers (“yes”, “no”, “I don’t know”). Respondents received one score for each correct answer while having no negative score for wrong answers. Respondent scores for all 10 knowledge questions were of 0-10. Attitudes were assessed using respondents’ answers as “strong” (score 2), “weak’ (score 1), and “null” (score 0). Therefore the total score of attitude range from 0-20. The Likert scale was checked on a 4 - point scale from ’always’ (score 3), “often” (score 2), “sometimes” (score 1), and ’never” (score 0) to asses the practice of subjects. The total scores on the 10 practice questions ranged from 0-30.

### 2.2 Statistical Analysis

Mean and standard deviation for knowledge, attitudes and practice were calculated. The Student’s t test was used to find out the difference between knowledge, attitude and practice among female and male students and also between year 6 and year 7 of students.

## 3. Results

Ninety two percent of subject responded to the questions. The scores of KAP are demonstrated in Table 1. The mean score of practice among women was higher than men (P<0.008) and medical students in year 7 had higher scores of knowledge than year 6 students (P<0.021). The most important reason for low score in practice was workload (48.6%), dry skin (6.8%) and inadequate equipment (44.6%). Percentage of respondents’ correct answers to KAP questions regarding standard precautions are demonstrated in table 2. The knowledge and practice of “hand washing after accidental contact” had highest correct responses (100% and 91.2% respectively), while the lowest percentage (50.8%) of correct answer in relation to knowledge was observed about “hand washing before and after using gloves” (Table 2). In relation to attitude, the highest and lowest rate of correct answer were shown in “wearing gloves when touching mucus membrane or non intact skin” (90.5%) and “hand washing before and after using gloves” (40.5%). The lowest percentage of correct answer (13.5%) was in practice of “washing before and after patient care”. In Table 3, distribution of participants’ answers in relation to knowledge, attitude and practice towards standard precautions are demonstrated. Among respondents, 20.9% did not have any knowledge toward needle bent before disposal.

**Table 1.** Knowledge, attitude and practice scores of medical students

	Knowledge	Attitude	Practice
**Sex**			
Female	7.1±1.5	17.1±2.9	18.7±4.6
Male	6.3±2.4	15.4±5.1	16.6±3.9
P	0.063	0.058	0.008

**Year of study**			
Year 6	6.5±2	16.2±4.3	17.9±4.8
Year 7	7.2±1.5	17±3	18.2±41
P	0.021	0.22	0.68

**Table 2:** Percentage of respondents’ correct answers to KAP questions regarding standard precautions

Questions	Knowledge (%)	Attitude (%)	Practice (%)
1. Hand washing before and after patient care	89.9	77.7	13.5
2. Hand washing before and after using gloves	50.8	40.5	16.2
3. Hand washing after accidental contact with blood, bloody fluid, secretions, contaminated item	100	98	91.2
4. Gloves should be worn when touching mucus membrane or non intact skin	90.5	90.5	58.1
5. Goggles should be worn to protect mucous membrane of the eyes	91.2	87.2	23.6
6. washing hands with Betadine after exposure to patients blood, bloody fluids, secretions or contaminated items	71.6	67.6	15.5
7. A surgical mask should be worn to protect the nose and mouth from invasive processor and activities	83.8	70.3	24.3
8. needle should be bent before disposal	62.1	56.7	14.2
9. Needle should be recap before disposal	80.4	79.7	60.8
10. gown should be worn when there is a risk of contaminated with aggressive processors and activities	71.6	63.5	19.5

**Table 3:** Distribution of participants’ answers in relation to knowledge, attitude and practice regarding standard precautions

**Questions**	**Knowledge (%)**			**Attitudes (%)**			**Practice (%)**			

	Yes	No	I don’t know	High	Low	never	always	often	sometime	never
1.Hand washing before and after patient care	89.9	10.1	0	77.7	16.2	6.1	13.5	33.8	45.3	7.4
2. Hand washing before and after using gloves	56.8	27.7	15.5	40.5	48.7	10.8	16.2	17.6	36.5	29.7
3. Hand washing after accidental contact with blood bloody fluid, secretions, contaminated item	1 0 0.0	0	0	98	2	0	91.2	4.7	4.1	0
4. Gloves should be worn when touching mucous membranes or non intact skin	90.5	5.4	4.1	90.5	5.4	4.1	58.1	29.1	7.4	5.4
5. Goggles should be worn to protect mucous membranes of the eyes	91.2	8.8	0	87.2	10.8	2	23.6	23.7	35.8	16.9
6. washing hands with betadine after exposure to patients blood bloody fluids, secretions or contaminated items	71.6	8.8	19.6	67.6	24.3	8.1	15.5	14.9	36.5	33.1
7. A surgical mask should be worn to protect the nose and mouth from invasive processors and activities	83.6	8.1	8.1	70.3	18.9	10.8	24.3	24.4	32.4	18.9
8. needle should be bent before disposal	62.1	16.9	20.9	56.7	23	20.3	14.2	17.5	29.1	39.2
9. Needle should be recap before disposal	80.4	15.5	4.1	79.7	13.5	6.8	60.8	17.6	11.5	10.1

## 4. Discussion

This survey described the knowledge, attitude and practice of medical students at Qazvin University of medical science in Iran. Our study revealed that while the knowledge and attitudes among medical students were acceptable but practices towards standard isolation precautions were poor as found in other studies (Askarian, 2007; Becker, 1990; Hersey, 1994; Mangione, 2007). It is expressed that, this disparity could be due to the unavailability of protective barriers, inadequate equipment, carelessness and malpractice of senior colleagues (Askarian, 2004). While In our study the most important reasons for this disparity were over workload, inadequate equipment and dry skin. Education is critical element in practice changes. This must be noticed and emphasize on it especially during a clinical practice. The occupational infection exposure risk is high in Iran. This is due to inadequate supply of personal protective equipment, improper disposal of medical waste and lack of effective needle disposal systems (Malekzadeh, 2001). Although 89.9% of the students had knowledge of ’hand washing before and after patient care”, but only 13.5% were practicing that. The student’s response to practicing of “hand washing after accidental contact with blood, bloody fluid, secretions and contaminated item” was 91.2%. We had limitations in this study. We did not have large group of students and also they were available for short duration in hospital clinical setting, where the study was conducted. These resulted in choosing small sample size and short duration of the study. In addition, we did not observe the practice of participant; however, the result of this study showed that in high risk situation the practice of medical students towards standard isolation was improved. The mean score of “practice” in female students was more than male students (P <0.008), while there were not any difference in their knowledge and attitude. This result revealed that, although in general, improving knowledge and attitude positively effect on practice and behavior, but it isn’t only predictors as seen in differentiation in mean score of knowledge in two groups of year 6 and year 7 and also male and female medical students (Table 1).

## 5. Conclusion

In conclusion, our results revealed that, the practice of standard isolation precautions among medical students of Qazvin University of Medical Sciences was poor. Having knowledge and positive attitude alone doesn’t influence practice. In addition the necessity of standard isolation in prevention of disease in patients in all duration of education must be emphasized and facilities should be improved. A gown should be worn when there is a risk of contamination with aggressive processor and activity
